# Correction: Time to diabetic neuropathy and its predictors among adult type 2 diabetes mellitus patients in Amhara regional state Comprehensive Specialized Hospitals, Northwest Ethiopia, 2022: A retrospective follow up study

**DOI:** 10.1371/journal.pone.0311424

**Published:** 2024-09-27

**Authors:** Sharie Tantigegn, Atsede Alle Ewunetie, Bekalu Endalew, Abiot Aschale, Muluye Gebrie, Gedefaw Diress, Moges Agazhe Assemie

The third and seventh author’s names are spelled incorrectly. The correct names are Moges Agazhe Assemie and Bekalu Endalew respectively.

The third and seventh authors are listed out of order. Please view the correct author order, affiliations, and citation here:

Sharie Tantigegn^1^, Atsede Alle Ewunetie^2^, Bekalu Endalew^2^, Abiot Aschale^2^, Muluye Gebrie^2^, Gedefaw Diress^2^, Moges Agazhe Assemie^2^

**1.** Dega Damot District Health Office, West Gojjam, Feresbet, Ethiopia, **2.** Department of Public Health, College of Health Sciences, Debre Markos University, Debre Markos, Ethiopia.

Tantigegn S, Ewunetie AA, Endalew B, Aschale A, Gebrie M, Diress G, et al. (2023) Time to diabetic neuropathy and its predictors among adult type 2 diabetes mellitus patients in Amhara regional state Comprehensive Specialized Hospitals, Northwest Ethiopia, 2022: A retrospective follow up study. PLoS ONE 18(4): e0284568. https://doi.org/10.1371/journal.pone.0284568

## References

[1] Tantigegn S, Ewunetie AA, Agazhe M, Aschale A, Gebrie M, Diress G, et al. (2023) Time to diabetic neuropathy and its predictors among adult type 2 diabetes mellitus patients in Amhara regional state Comprehensive Specialized Hospitals, Northwest Ethiopia, 2022: A retrospective follow up study. PLoS ONE 18(4): e0284568. doi: 10.1371/journal.pone.0284568 37115732 PMC10146479

